# Co-development of pyogenic granuloma and capillary hemangioma on the alveolar ridge associated with a dental implant: a case report

**DOI:** 10.1186/1752-1947-8-192

**Published:** 2014-06-16

**Authors:** Young-Hoon Kang, June-Ho Byun, Mun-Jeong Choi, Jong-Sil Lee, Jung-Hui Jang, Young-Il Kim, Bong-Wook Park

**Affiliations:** 1 Department of Oral and Maxillofacial Surgery, Institute of Health Science, School of Medicine, Gyeongsang National University, Jinju, Korea; 2 Department of Pathology, School of Medicine, Gyeonsang National University, Jinju, Korea; 3 Hanil Dental Clinic, Jinju, Korea

**Keywords:** Antithrombotic therapy, Capillary hemangioma, Dental implant, Pyogenic granuloma

## Abstract

**Introduction:**

The development of various benign oral mucosal lesions associated with dental implants, such as pyogenic granuloma or peripheral giant cell granuloma, has been rarely reported. However, the occurrence of vascular diseases, such as hemangioma, related to dental implants has not been explored in the literature. In this study, we report a case of co-development of pyogenic granuloma and capillary hemangioma on the alveolar ridge associated with a dental implant in a patient undergoing antithrombotic therapy. To the best of our knowledge, this is first case of hemangioma formation associated with a dental implant.

**Case presentation:**

A 68-year-old Korean man was referred for intermittent bleeding and a dome-shaped overgrowing mass on his upper alveolar ridge. He underwent dental implantation 5 years ago, and was started on warfarin for cerebral infarction a year ago. He had experienced gum bleeding and gingival mass formation 6 months after warfarinization; then, his implant fixture was removed. However, his gingival mass has been gradually increasing. The gingival mass was surgically excised, and revealed the coexistence of pyogenic granuloma and capillary hemangioma in histological analysis of the specimen. The lesion has showed no recurrence for more than a year.

**Conclusions:**

Regarding immunostaining features, the endothelial cell markers, CD34 and CD31, and the mesenchymal cell marker, vimentin, were strongly detected, but cell proliferation marker, Ki-67, was negatively expressed in the endothelial cells of the hemangioma portion. However, in the pyogenic granuloma portion, CD34 was almost negatively detected, whereas vimentin and Ki-67 were highly detected in the fibroblast-like tumor cells. According to these heterogeneous characteristics of the lesion, the patient was diagnosed with coexistence of pyogenic granuloma and capillary hemangioma associated with the dental implant on the attached gingiva. We recommend that patients with dental implants who have chronic peri-implantitis under antithrombotic therapy should be closely followed to ensure early detection of oral mucosal abnormalities.

## Introduction

Dental implantation has become one of the most common dental procedures. However, some serious complications related to dental implant placement, including hemorrhage and life-threatening swelling, have occasionally been reported [1]. In addition, the unexpected development of benign mucosal diseases, such as pyogenic granuloma (PG) or peripheral giant cell granuloma, that are associated with dental implants has been reported in several cases [2-6]. The etiology of these mucosal diseases related with dental implants has not been determined; however, chronic irritation due to corrosion of the fixture surface and chronic inflammation may be considered contributing factors to their pathogenesis [3].

In medically compromised patients, some unpredicted complications can be caused by simple dental procedures, such as tooth extraction or dental implant placement. In particular, antithrombotic therapy could induce postoperative bleeding or hematoma formation. However, the development of any vascular disease, including hemangioma or vascular malformation, in patients undergoing antithrombotic therapy has not been explored in the literature. Hemangioma is a benign tumor of vascular endothelial cells, characterized by the benign proliferation of blood vessels, and is the most common type of childhood tumor [7,8]. This particular vascular disease has also not been reported in relation with dental implant procedures.

In this study, we report a case of co-development of PG and capillary hemangioma in the upper alveolar ridge associated with a dental implant in a warfarinized patient. To confirm the characteristics of the lesion, the excised specimen was immunohistochemically analyzed with various antibodies for markers of endothelial cells, mesenchymal cells and cell proliferation. To the best of our knowledge, this is first case of hemangioma formation associated with a dental implant in a medically compromised patient.

## Case presentation

**Figure 1:**
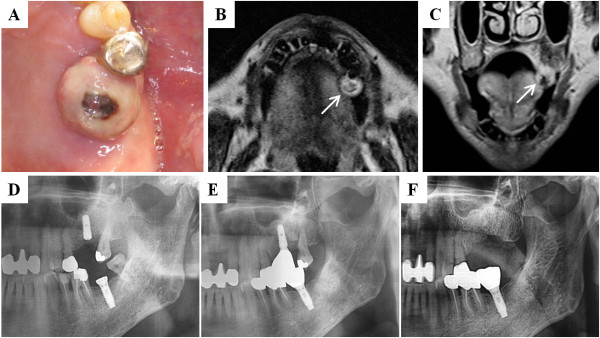
**Preoperative clinical and magnetic resonance imaging views (A-C) and follow-up panoramic views for implantation (D-F). (A)** A firm and round dome-shaped mass was observed in the site of the previous implant, the left maxillary first molar. **(B and C)** In magnetic resonance imaging, a 1.5cm heterogeneous mass was observed on the attached gingiva (arrows). **(D and E)** Panoramic views at 3 months **(D)** and 4 years **(E)** after fixture placement. **(E)** Progressive alveolar bone destruction due to peri-implantitis was observed at 4 years after implantation. **(F)** Panorama at the first visit to our clinic, 5 months after fixture removal. There is no associated bony defect with the gingival mass.

A 68-year-old Korean man was referred by his dentist for intermittent bleeding and a dome-shaped overgrowing mass on his upper left alveolar ridge (Figure 1A). He underwent dental implantation on his upper first molar site 5 years ago, and regular curettage has been done for the treatment of recurrence of peri-implantitis (Figures 1D and 1E). Four years after his dental implantation, he had a cerebral infarction and was started on antithrombotic therapy with warfarin by a neurologist. After 6 months of warfarinization, he experienced intermittent gum bleeding and an overgrowing gingival mass in the implant site. Thus, the implant was removed by his dentist for fixture mobility at 5 months before visiting our clinic. However, the gingival mass has been gradually increasing, and spontaneous gingival bleeding was encountered, even after the removal of the implant fixture.An intra-oral examination of the patient revealed a firm dome-shaped mass on his upper left alveolar ridge, which is the site of previous implantation. Although the center of the lesion was bluish, which was probably due to discoloration from occlusion trauma, other parts of the mass showed a pink-colored smooth surface (Figure 1A). Magnetic resonance imaging showed a 1.5cm round nonhomogeneous lesion on his upper left alveolar ridge, which was most probably a benign vascular neoplasm (Figure 1B and C). The lesion was surgically excised under local anesthesia, and complete coagulation on the surgical bed was obtained with electrocauterization.

**Figure 2:**
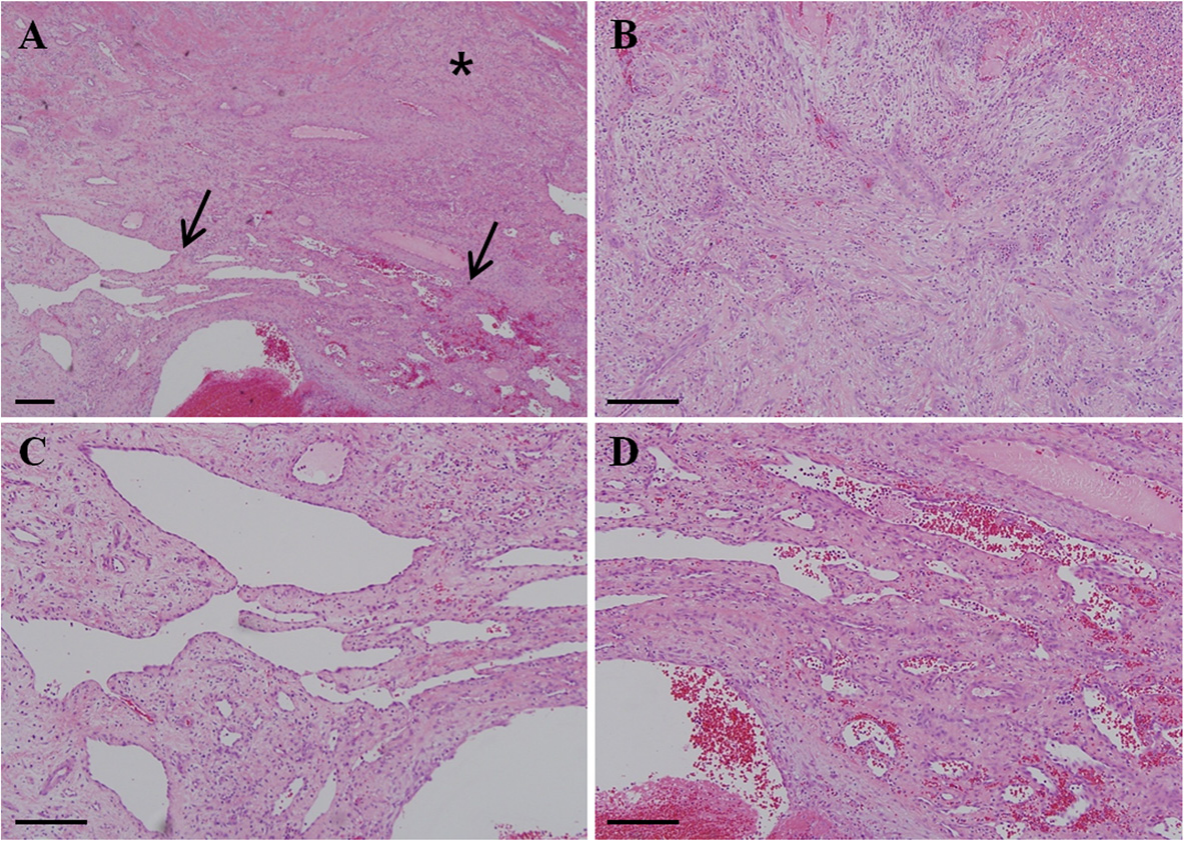
**Hematoxylin and eosin staining features of specimen. (A)** Tumor specimen at low magnification. Asterisk (*) indicates the pyogenic granuloma portion of the lesion, and arrows show the development of capillary hemangioma and thrombosis. **(B)** The pyogenic granuloma portion at higher magnification. Edematous granulation tissues and numerous small blood vessels with neutrophil infiltration were observed under the epithelium of the tumor. **(C and D)** Capillary hemangioma portion of the tumor at higher magnification showing numerous newly generated blood vessels filled with thrombi. Newly developed capillary vessels were seen to communicate with each other and lined with a thin endothelial cell layer. Scale bar = 200μm.

**Figure 3:**
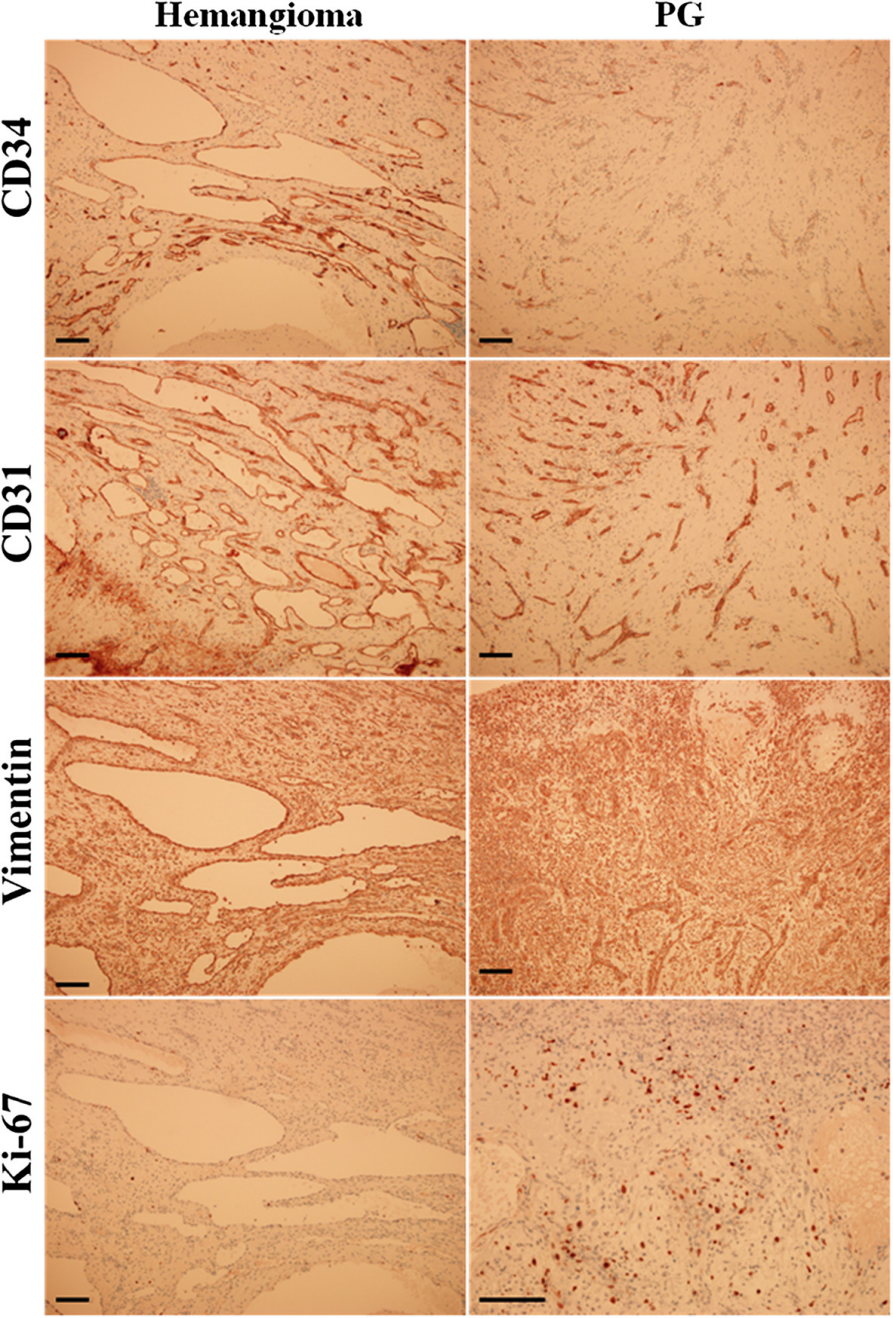
**Immunohistochemical analysis for the markers of endothelial cells, mesenchymal cells and cell proliferation in the specimen.** In the hemangioma portion of the lesion, the endothelial cell markers, CD34 and CD31, and the mesenchymal cell marker, vimentin, were strongly observed, but the cell proliferating marker, Ki-67, was negatively expressed in the newly developed vascular vessels. However, in the PG portion, CD34 was almost negatively detected, whereas vimentin and Ki-67 were highly detected in the fibroblast-like tumor cells. Scale bar = 100μm. CD31, Cluster of differentiation 31 (platelet endothelial cell adhesion molecule); CD34, Cluster of differentiation 34; Ki-67, MKI67; PG, pyogenic granuloma.

Histology of the specimen revealed an ulcerated nodule and edematous granulation tissues under the epithelium of the tumor with numerous small blood vessels and neutrophil infiltration. The patient was diagnosed with PG (Figures 2A and 2B). The deeper part of the lesion showed numerous newly developed vessels filled with thrombi and their communications with delicate fibrillar connective tissues, indicating capillary hemangioma (Figures 2A, 2C and 2D). To confirm the characteristics of the tumor, the slides were immunostained with specific marker antibodies for endothelial cells, mesenchymal cells, and cell proliferation. For immunohistochemical analysis, tumor specimens were embedded in a paraffin block, cut into 4μm sections and mounted on glass slides. The sections were maintained at room temperature for 12 hours and deparaffinized. After hydration, immunohistochemical staining was conducted using an automated immunostainer (BenchMark XT, Ventana Medical Systems Inc., Tucson, AZ, USA). The primary antibodies used and immunohistochemical staining results are summarized in Table 1.Regarding immunostaining features, vascular endothelial cell and hematopoietic progenitor markers, CD31 and CD34, were strongly expressed in neovascularized endothelial cells of the hemangioma portion. However, in PG, CD31 was highly detected in the small blood vessels of the tumor, whereas CD34 was rarely expressed. The mesenchymal cell marker, vimentin, was strongly detected in both hemangioma and PG portions. The cell proliferation marker, Ki-67, was almost negative in the hemangioma portion, but it was moderately expressed in the fibroblast-like tumor cells of the PG portion (Figure 3). According to these heterogeneous characteristics of the lesion, the patient was diagnosed with coexistence of PG and capillary hemangioma associated with the dental implant on the attached gingiva. The lesion has showed no recurrence or bleeding for more than a year.

**Table 1:** Primary antibodies and their dilution rate, and result of semi-quantitative analysis for immunostaining intensity

**Antibody**	**Dilution**	**Source**	**Immunostaining intensity**	
			**Hemangioma**	**PG**
**CD34**	1:3000	Neomarkers	**+++**	**–**
**CD31**	1:20	Dako	**+++**	**++**
**Vimentin**	1:20	Dako	**+++**	**+++**
**Ki-67**	1:2000	Dako	**–**	**++**

Abbreviations: CD31, Cluster of differentiation 31 (platelet endothelial cell adhesion molecule); CD34, Cluster of differentiation 34; Ki-67, MKI67; PG, pyogenic granuloma. Positive immunostaining intensities were graded as +++, ++, + and – for strong, moderate, weak and negative staining, respectively.

## Discussion

The causes of reactive peri-implant mucosal diseases have not been completely understood. One putative cause is the corrosion of the fixture surface and metal particles [3]. In addition, chronic mucosal inflammation resulting from poor oral hygiene or trauma from mobilized suprastructure of the implant could be considered contributing factors for peri-implant mucosal diseases [2,9]. It is well known that inflammatory cells facilitate the initial steps of tumorigenesis or, alternatively, inflammatory cells could be coopted by neoplastic cells during tumor progression [10,11]. In the present case, chronic inflammation due to recurrent peri-implantitis could be considered one of the contributing factors to the development of PG in the peri-implant gingival tissue.

Hemangioma is one of the most common tumors in neonates and children, and is classified into capillary and cavernous types on the basis of the size of vascular spaces and histology. Capillary hemangioma is characterized by numerous proliferating small thin-walled blood-filled vessels composed of a single layer of endothelial cells, surrounded by a discontinuous layer of pericytes and reticular fibers, and by its uncommonness in older aged people [7,8]. However, the occurrence of capillary hemangioma related to dental implants or antithrombotic therapy has not been explained in the literature. On the contrary, antithrombotic therapy with heparin or warfarin has been used as complementary therapy in patients with cancer because of its anti-angiogenic property in tumors, which results in the interference of tumor cell proliferation and metastasis processes [12-14]. These actions are mainly done by the binding of antithrombotic agents to angiogenic growth factors, such as fibroblast growth factors and vascular endothelial growth factors, which inhibit angiogenesis [12]. However, the most common side effect of warfarin is hemorrhage, even though the risk of life-threatening severe bleeding is not large. Interestingly, hemorrhage shows a relatively higher frequency in the gum or nose than in other organs [15]. In addition, the risk of bleeding is increased when warfarin is combined with antiplatelet drugs or nonsteroidal anti-inflammatory drugs [16], or in elderly patients [17].

In this report, the patient had used the prosthesis after implantation for 4.5 years, and undertaken antithrombotic therapy with warfarin for a year. He felt an abnormal gingival mass formation and spontaneous gum bleeding starting 6 months after warfarinization. Interestingly, the gingival mass showed two different entities, PG and capillary hemangioma, in terms of its histological features. In the immunohistochemistry of the hemangioma portion of the lesion, hematopoietic markers, CD31 and CD34, were highly detected in neogenerated vascular tissues, whereas the cell proliferating marker, Ki-67, was negatively detected in the endothelial cells of the hemangioma. However, almost negative expression of CD34 but strongly detected Ki-67 was observed in the PG portion. The mesenchymal cell marker, vimentin, was strongly detected in both endothelial cells of the hemangioma and fibroblasts in the PG portion. The results of the immunohistochemical analysis of the present case indicate that these histomorphologically different entities possess different properties and have developed coincidentally in one gingival mass.

## Conclusions

Although a direct relationship between hemangioma development and warfarinization could not be established in this case, frequent bleeding due to antithrombotic therapy and chronic inflammation of peri-implantitis are suspected to be causally associated with the development of neovascularization as well as PG in the gum tissue. Chronic irritation, including inflammation of peri-implantitis, could generate various neoplasms in the oral mucosa [18,19]. To the best of our knowledge, this is the first case of co-development of PG and capillary hemangioma in the attached gingiva associated with a dental implant in a medically compromised patient. We recommend that patients with dental implants who have chronic peri-implantitis under antithrombotic therapy should be closely followed to ensure early detection of oral mucosal abnormalities. In addition, it is advisable to report all clinical cases of lesions associated with dental implants in order to contribute information that will help to determine the etiology, pathogenesis, and course of these lesions.

## Consent

Written informed consent was obtained from the patient for publication of this case report and accompanying images. A copy of the written consent is available for review by the Editor-in-Chief of this journal.

## Competing interests

The authors declare that they have no competing interests.

## Authors’ contributions

YHK collected and analyzed the patient data, and was a partial contributor in writing the manuscript. JHB and MJC assisted treatment of the patient and performed patient care. JSL evaluated the histological and immunohistochemical results of the tumor specimen. JHJ and YIK referred the patient to our clinic and offered the old data when the patient had undertaken dental implantation. BWP performed the patient management, and was a major contributor in writing the manuscript. All authors read and approved the final manuscript.
